# Systematic Evaluation of the T30 Neurostimulator Treatment for Tinnitus: A Double-Blind Randomised Placebo-Controlled Trial with Open-Label Extension

**DOI:** 10.3390/brainsci12030317

**Published:** 2022-02-26

**Authors:** Deborah Ann Hall, Robert Henryk Pierzycki, Holly Thomas, David Greenberg, Magdalena Sereda, Derek James Hoare

**Affiliations:** 1 NIHR Nottingham Biomedical Research Centre, Ropewalk House, 113 the Ropewalk, Nottingham NG1 5DU, UK; deborah.hall@hw.ac.uk (D.A.H.); rpierzycki@gmail.com (R.H.P.); ht6799@hotmail.co.uk (H.T.); magdalena.sereda@nottingham.ac.uk (M.S.); 2 Hearing Sciences, Mental Health and Clinical Neurosciences, School of Medicine, University of Nottingham, Nottingham NG7 2UH, UK; 3 School of Social Sciences, Heriot-Watt University Malaysia, No. 1, Jalan Venna P5/2, Precinct 5, Putrajaya 62200, Malaysia; 4 Department of Ear, Nose and Throat (ENT), Nottingham University Hospitals (NHS) Trust, Queen’s Medical Centre Campus, Derby Road, Nottingham NG7 2UH, UK; 5 Ear Institute, University College London (UCL), 332 Gray’s Inn Road, London WC1X 8EE, UK; david@eave.io

**Keywords:** sound therapy, neuromodulation, neural synchrony, acoustic CR neuromodulation, tinnitus disorder, quality of life

## Abstract

Tinnitus is often triggered by cochlear damage and has been linked with aberrant patterns of neuronal activity. Acoustic Coordinated Reset (CR^®^) Neuromodulation is a sound therapy hypothesised to reduce tinnitus symptoms by desynchronising pathological brain activity using a portable acoustic device (the T30 neurostimulator). We report results of a pivotal trial to test the efficacy of this intervention. This two-centre, double-blind randomised controlled trial with long-term open-label extension, was undertaken between February 2012 and February 2014 in the UK. Participants were 100 adults with tinnitus as a primary complaint, recruited through hearing clinics and media advertisements. Intervention was the device programmed either with the proprietary sound sequence or placebo algorithm, fit by one of five trained audiologists. Minimisation software provided group allocation (1:1 randomisation), with groups matched for age, gender, hearing loss and tinnitus severity. Allocation was masked from participants and assessors during the trial. The primary measure of efficacy was change in tinnitus symptom severity between groups, measured using the Tinnitus Handicap Questionnaire at 12 weeks. Secondary outcomes were other measures of tinnitus symptom severity, health-related quality of life, and perceptual characteristics (pitch, loudness, bandwidth) at 12 weeks, and Tinnitus Handicap Questionnaire at 36 weeks (open-label extension). A statistician blinded to the allocation conducted an intention-to-treat analysis that employed linear regressions on minimisation variables, trial centre and intervention group, with multiple imputations for missing data. The study was registered on clinicaltrials.gov (NCT01541969). We screened 391 individuals and assigned interventions to 100 eligible participants. The primary outcome was not statistically significant between groups (mean group = −0.45, 95% CI −5.25 to 4.35; *p* = 0.85), nor were any of the secondary outcomes. Four adverse events occurred during the trial. Analysis of tinnitus symptom severity data collected across the 24-week open-label extension showed no statistically significant within-group changes after 12, 24, or 36 weeks treatment with the proprietary sound sequence. While individual participants may benefit from sound therapy, Acoustic CR^®^ Neuromodulation did not lead to group-mean reductions on tinnitus symptom severity or other measures compared to placebo, or over time.

## 1. Introduction

The chronic experience of tinnitus affects about 16% of adults [1] with estimates ranging 6–42% in children and adolescents [2]. For many it is a chronic and debilitating condition, recently coined as ‘tinnitus disorder’ [3] causing problems with sleep, concentration, relaxation, emotional and mental wellbeing, and overall quality of life [4]. Treatments such as cognitive behavioural therapy (CBT), hearing aids, and sound generators have demonstrated value in reducing tinnitus symptom severity, reducing awareness of symptoms, and improving quality of life for some patients [5,6]. However, no current treatment demonstrably eliminates the percept of tinnitus.

There is consensus that the percept of tinnitus is triggered by cochlear damage which degrades the auditory input to central neural pathways. This initiates plastic changes within the auditory brain that result in aberrant patterns of neuronal activity interpreted as tinnitus sound. Large-scale studies have established hearing loss as a major risk factor for tinnitus. For example, Nondahl et al. [7] reported that hearing loss increased the odds of reporting tinnitus by a factor of 3.20 (95% CI = 2.33 to 4.38), and that this effect was independent of age and sex. A comparable association (OR = 2.31, 95% CI = 1.46 to 3.66) was identified by Gopinath et al. [8] in an Australian population.

Many of those with tinnitus have hearing loss that developed from a high-frequency sensorineural hearing damage through noise exposure, otological disease, or the ageing process [7,8,9]. Likely candidate mechanisms for the underlying pathology have been investigated using animal models of noise-induced cochlear damage. Noise-induced hearing loss shows both immediate and long-term decreases in spontaneous firing rate in the auditory nerve, contrasted with increases in neural activity in primary auditory cortex, cochlear nucleus, and inferior colliculus [10]. This effect could reflect a homeostatic rescaling of neuron input/output functions to stabilise neuronal activity after hearing loss [11,12]. Increased temporal synchrony in neural firing has also been observed in neurons with characteristic frequencies in the affected region even without concomitant increases in spontaneous firing rate [12]. This change is closely confined to the hearing loss region and appears to reflect hypersynchronous network activity over lateral connections which develop when the constraints of intracortical inhibition are weakened [13]. Tinnitus may thus be the perceptual consequence of stabilised hypersynchrony over large-scale neural assemblies.

A computational model of this aberrant synchrony was developed by Tass and Popovych [14], which predicts aberrant activity patterns might be reduced through acoustic stimulation. Specifically, they proposed a desynchronising sound sequence to interrupt and ‘unlearn’ the pathological synchronous network activity. According to the model, the therapeutic sound sequence could lead to a permanent reduction of tinnitus by promoting a transition to a stable state of asynchronous spontaneous activity through plasticity [14]. This process is known as ‘coordinated reset’ (CR^®^). CR was developed initially for therapeutic electrical deep brain stimulation in Parkinson’s disease [15] and subsequently translated into the acoustic domain. The parameters of an effective desynchronising sequence have been explored in previous work [15,16,17,18]. Computational simulations indicate that the random variation of the stimulation sequence, the ON–OFF pattern of stimulation and silence cycles and the cycle rate can optimise the desynchronising CR effect.

An initial proof-of-concept/dose finding phase 1 study (RESET1; *clinicaltrial.gov* ID: NCT00927121) assessed safety of this treatment approach and provided an indication of effects of four different desynchronising sound sequences for people with tinnitus [19]. One of the therapeutic sounds (Group 1) was tailored to each patient based on their dominant tinnitus pitch (fT) with two tones placed above and two below that frequency between 0.5 fT and 2.0 fT, and a 3:2 ON–OFF pattern presented at a four-tone cycle rate of 1.5 Hz. Participants were recommended to wear a portable acoustic device (the T30 neurostimulator) for 4–6 h per day every day applied either continuously or in several sessions of at least 1 h each to utilise cumulative effects. The authors also indicated that stimulation frequencies could be readjusted if fT changed between clinic visits. Sixty-three participants, divided across five groups, were fitted with a device. Four groups were allocated different versions of a four-tone sequence of tones selected relative to the tinnitus pitch, variably predicted to disrupt hypersynchronous neuronal activity, and promote an asynchronous spontaneous state. The fifth group received a placebo tone sequence containing low frequency tones that excluded the tinnitus frequency region. There were 13 adverse events (AEs) during a 12-week blinded study phase (e.g., transient headache, transient increase of tinnitus loudness and annoyance) none of which were deemed to be severe. Within groups, reduction in global tinnitus severity (Tinnitus Questionnaire; TQ) [20] was statistically significant for all intervention groups (mean reductions in TQ scores for the intervention groups 1–4 were between 5.2 and 15.5 points), but not for the placebo group (G5; reduction was 8.4 points). However, as this was a proof-of-concept study between group comparisons of change in TQ scores were not analysed.

Following proof-of-concept, non-randomised, non-controlled open-label trials and various exploratory analyses were published (see [21] for a systematic review). Trials included a retrospective study with 66 patients [22], and a prospective registered study (*clinicaltrials.gov* ID: NCT01435317) with 200 patients [23]. Both studies prescribed the T30 neurostimulator in the context of acoustic CR^®^ neuromodulation therapy. Studies reported alleviation of tinnitus after 6 and 12 months of therapy, respectively, but as they were not controlled effects cannot be directly or wholly attributed to Acoustic CR^®^ Neuromodulation.

Most recently, a randomised non-inferiority trial compared acoustic CR^®^ neuromodulation (DesyncraTM) to a short-form CBT ([24], *clinicaltrials.gov* ID: NCT03022084). DesyncraTM involved 4–6 h daily use of the device over 24 weeks. CBT involved six therapist sessions of 1 h to balance the amount of clinical contact in each group. Randomisation included stratification regarding hearing aid use, and the primary outcome, tinnitus distress, was measured using the TQ. The trial concluded non-inferiority of the T30 neurostimulator treatment [24] (i.e., outcomes were not unacceptably worse than those for CBT). Ancillary benefits were not assessed.

Here, we report the findings from the first randomised controlled trial (RCT) to test the efficacy of treatment using the T30 neurostimulator. The aim of this trial was to determine whether the desynchronising sound sequence delivered by the T30 neurostimulator significantly reduced tinnitus symptom severity compared to an active placebo control sound sequence. Secondary outcomes evaluated whether treatment significantly changed tinnitus symptom severity, quality of life, or tinnitus percept, compared to a placebo control.

## 2. Materials and Methods

### 2.1. Trial Design

This was a two-centre, double-blind, placebo-controlled, parallel-group (with 1:1 randomisation) study conducted in the UK (called RESET2). The trial had two phases: a 12-week RCT, followed by a 24-week open-label long term extension (LTE). Our reporting follows standards defined in the Consolidated Standards of Reporting Trials (CONSORT) Statement [25].

The protocol was co-developed by authors, with expert opinion provided by the developer of Coordinated Reset (P. Tass) and the manufacturer of the T30 neurostimulator at the time (Adaptive Neuromodulation GmbH) [26]. The protocol was independently reviewed and approved by the National Research Ethics Service (NRES) Committee, East Midlands—Nottingham 1 (Ref 12/EM/0025). The trial sponsor was the Nottingham University Hospitals National Health Service (NHS) Trust, Research and Innovation Department (Ref 11IH006). An agreement was previously held between the sponsor and funder to embargo journal publication resulting in the delay between completion of the trail and journal publication. However, the main outcomes were made available on clinicaltrials.gov on 18 June 2015.

### 2.2. Participants

Inclusion and exclusion criteria are given in Table 1. Participant recruitment was via public response to an advert placed on the website of the lead author’s organisation and in local hearing clinics, as well as publicity by the national media. Those interested contacted the team for a participant information sheet which described the study and were later telephoned to screen for potential eligibility (questions on general health, otology comorbidities, hearing aid usage, and willingness to comply with trial requirements). People who passed this screening then visited their preferred study centre for a 2–3-h detailed assessment (visit 1) to determine suitability for the intervention, and eligibility for the trial. Assessment involved otoscopy, pure tone audiometry (PTA), Tinnitus Case History Questionnaire (TCHQ; [27]), Tinnitus Handicap Inventory (THI; [28]), tinnitus pitch and loudness estimates using the Tinnitus Tester [29,30], and the Beck Anxiety (BAI) and Depression (BDI-II) Inventories [31]. Participants were also required to refrain from starting any new treatments for tinnitus during the trial period. There was no reimbursement for participation, but participants could keep the device at the end of the trial. Informed written consent was obtained at the visit 1 assessment.

### 2.3. Study Settings

The trial took place at the National Institute for Health Research (NIHR) Nottingham Hearing Biomedical Research Unit (BRU) [now NIHR Nottingham Biomedical Research Centre] and the University College London (UCL) Ear Institute from February 2012 to February 2014. Both centres are based in cities with a large catchment area that includes a wide population demographic. Of the 100 participants allocated to one of the two groups, 55 participated at the Nottingham centre and 45 at the London centre.

### 2.4. Interventions

Participants in treatment and placebo groups received the same T30 neurostimulator device and headphones at visit 2, the only difference being the sound prescription. For the treatment group, prescription was the same as for Group 1 in RESET1 [19] insofar as it included two tones above and two tones below the matched tinnitus pitch (i.e., frequency range: 0.5 fT: 2.0 fT). Tones were presented randomly at a cycle repetition rate of 1.5 Hz, i.e., in the lower δ frequency range of oscillatory brain activity, because the primary target for desynchronisation is pathological δ band activity. For the placebo group, the four-tone stimulation included frequencies in the 500 to 4000 Hz range only, but the tinnitus frequency region was excluded (fT/2, fT*2), and the four-tone cycle rate was 0.3 Hz (five times slower than treatment).

**Figure 1:**
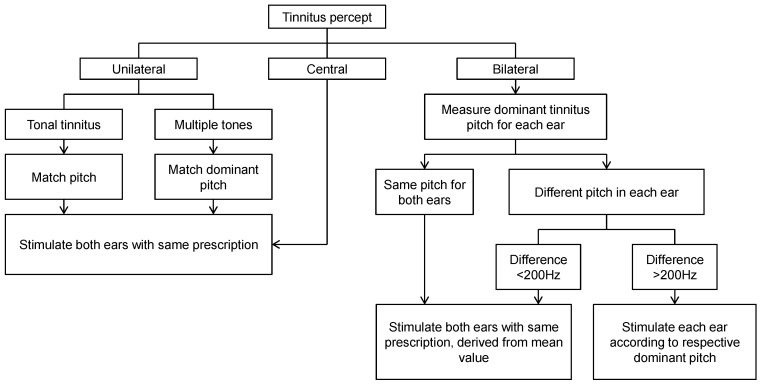
Overview of the T30 neurostimulator device fitting protocol used by the trial audiologists. A central tinnitus refers to a unified percept that is experienced in the head (not in the ear). In this case, only one ear needs to be assessed.

At both centres, audiologists were trained to fit the T30 neurostimulator using a prescription protocol that was developed in cooperation with The Tinnitus Clinic and Adaptive Neuromodulation GmbH. It provided guidelines for dealing with a range of potential clinical scenarios (such as unilateral tinnitus or differences in tinnitus percept across the two ears) (Figure 1). Dominant pitch was ascertained using an adaptive bracketing method with pure tones presented at step sizes of 500 Hz from 500 to 12,000 Hz in ascending and descending sweeps. The bracket was informed by participant responses to these sweeps describing frequencies that bordered their own tinnitus percept, from which a two-alternative forced-choice method was used to eventually identify the dominant tinnitus pitch. Once a dominant pitch had been identified, the manufacturer’s proprietary software set the sound prescription on the participant’s device. The loudness of each of the four tones was adjusted by the participant to be perceived as equally loud to one another and presented at a soft but audible listening level. Each device was set with a 20 dB volume control (+/−10 midpoint). All participants were prescribed with bilateral stimulation delivered through ear bud headphones and were given accessories including ear tips and cleaning tools. Participants were instructed to use the device between 4 and 6 h every day for 12 weeks. During this period, they maintained a daily diary to account hours of usage and whether usage was in a quiet or noisy environment. All participants were given the same information about the device and expected trial outcomes, irrespective of their group allocation.

The device prescription was reassessed by the audiologist at scheduled visits during the 12-week RCT and these corresponded to visits 3, 4, 5, and 6. During the 24-week open-label extension device prescription was reassessed at scheduled interim visits corresponding to visits 7, 8, 9, and 10 for the placebo-crossover group, and 8, 9 and 10 for the intervention group. Whenever there was a change in dominant tinnitus pitch, the prescription was adjusted accordingly. When the dominant tinnitus pitch increased beyond the output limits of the device (i.e., >10 kHz), the participant paused the treatment, and was invited back for reassessment 2 weeks later. If the dominant tinnitus pitch was outside of the 0.2 to 10 kHz frequency range at reassessment, then the participant was withdrawn from the trial.

At the end of the 12-week RCT period, participants and audiologists were unblinded to group allocation. The T30 neurostimulator belonging to each participant in the placebo group was reprogrammed with a console code that undid any previous settings, resetting the device with the verum stimulation algorithm. These participants were instructed to use the device between 4 and 6 h every day for the 12 weeks, and 4 h per day thereafter. Participants in the treatment group were advised to wear the device for 4 h daily during the open-label LTE.

### 2.5. Outcomes

The primary outcome with respect to clinical efficacy was the difference between groups in the mean change from baseline (visit 2) to 12 weeks (visit 6) in tinnitus symptom severity, as measured by the Tinnitus Handicap Questionnaire (THQ) global score [32]. THQ was selected because it constitutes one of the patient-reported outcome measures with better psychometric properties and some prior evidence on which to estimate sample size to detect treatment-related change [33]. It contains 27 statements addressing a range of issues encompassing subscales for (1) social, emotional, and behavioural effects of tinnitus, and (2) hearing. Participants were instructed to rate how much they agreed or disagreed with each statement by placing a mark on a scale from 0 (strongly disagree) to 100 (strongly agree). Global scores are scaled from 0–2700 to 0–100, with higher global scores indicative of more substantial impact on everyday life. As a secondary outcome, the THQ was also recorded at week 24 (visit 9) for participants in the placebo group (so that we could pool with the 12 weeks data from the treatment group), and at week 36 (visit 10) for all participants, compared to baseline (visit 2).

Further secondary outcomes included additional measures of tinnitus symptom severity, health-related quality of life, psychoacoustic measures of tinnitus percept, and resting state EEG. Resting-state EEG has been reported in a previous publication [34]. Tinnitus symptom severity was measured using two additional questionnaires, the THI and Tinnitus Functional Index (TFI) [35]. The THI was chosen as it is the most frequently used questionnaire for diagnosis in the UK [36] and so provides a familiar clinical benchmark even though it was not developed specifically to measure change over time. The THI is easy to administer and score with 25 questions on thoughts and feelings about tinnitus, each with three response options (yes = 4, sometimes = 2, no = 0). The TFI was developed for assessing the overall functional impact of tinnitus and any treatment-related change. Again, the TFI is easy to administer and score with 25 items requiring participants to rate on a 10-point scale their tinnitus over the past week. For both tinnitus questionnaires, the global score reflects the sum of all responses from 0 to 100, with a higher global score indicative of greater severity of tinnitus symptoms. The TFI includes a measure of tinnitus loudness (item 2) and a measure of tinnitus annoyance (item 3), providing for a post hoc comparison with the visual analogue scale (VAS) data reported in RESET1.

Quality of life was assessed by the WHOQOL-BREF [37]. The 26 items in the WHOQOL-BREF ask participants to rate their feelings within last four weeks on a 5-point scale from very poor to very good, with question 1 asking about an individual’s overall perception of quality of life. Responses also provide information on four domains related to quality of life (physical health, psychological, social relationships, and environment), with transformed scores scaling from 0 to 100.

Three psychoacoustic measures of tinnitus percept were loudness, dominant pitch, and bandwidth, measured using the Tinnitus Tester software [29,30] installed on a PC connected to a Tucker Davis Technologies programmable attenuator and driver unit for a set of Sennheiser HDA200 headphones. In the loudness matching procedure, participants adjusted the level of 11 sound tokens centred at frequencies from 0.5 to 12 kHz to be equally loud to their tinnitus percept. Tinnitus loudness was defined as the matched attenuation value at the centre frequency of 1 kHz. To estimate dominant pitch and bandwidth, the tinnitus spectrum was plotted using ratings of the likeness of the same 11 sound tokens to the pitch of their tinnitus using a 100-point scale. For example, the dominant tinnitus pitch corresponded to the sound frequency with the highest likeness rating. The tinnitus bandwidth was also calculated by weighting each frequency by its percentage likeness rating and taking the standard deviation across the 11 weighted test frequencies [38].

Outcomes were administered by a trained researcher at each centre. The THQ, THI, TFI, WHOQOL-BREF, and the Tinnitus Tester were administered at the eligibility assessment (visit 1), and again at a baseline assessment used for the efficacy analyses (visit 2). For the RCT, scheduled interim visits were expected at 2 weeks (visit 3), 4 weeks (visit 4), 8 weeks (visit 5), and 12 weeks (visit 6) from the date of the device fitting. No outcomes were collected at visit 3 and at the other visits the outcomes were always assessed before the audiologist appointment for device fitting and adjustment. Visits 2, 4, 5 and 6 obtained outcomes data for the THQ, TFI, and Tinnitus Tester. Outcome data for the WHOQOL-BREF and THI were obtained only at visits 2 and 6. For the open-label LTE, THQ and TFI were obtained at visits 9 and 10.

### 2.6. Sample Size

THQ data from a published study of tinnitus masking [39] were used to estimate the required sample size (two-sample *t*-test power analysis) performed in R [40]. After a 12-week intervention, Henry et al. [39] found that a difference in mean THQ score of 194 between groups, with a pooled standard deviation (SD) of 450 was significant and represented a medium effect size. We assumed a difference in mean THQ score of 250 between groups with a pooled SD of 425 as representative of a large effect size. Thus, for a two-sided significance level of 0.05 and 80% power, it was estimated that 47 participants were required for each group. We predicted a drop-out rate of approximately 5% in 12 weeks and therefore 100 participants were recruited.

For analysis, we scaled THQ scores from a possible range of 0–2700 to 0–100. This has no impact on the outcome of statistical analysis but is preferable because it facilitates comparison across THI and TFI which also have the range 0–100. If a medium effect size, indicative of a clinically meaningful change, reflects a difference in mean THQ of 194 points, then the scaled score equivalent would be 7.19.

### 2.7. Interim Analysis and Stopping Rules

There were no planned interim analyses or stopping rules for the trial.

### 2.8. Randomisation and Blinding

Randomisation was performed using minimisation (MINIM) software [41]. Minimisation achieved matching between the treatment and placebo groups for age, gender, hearing loss (average of PTA at 0.5, 1, 2, and 4 kHz) and grade of tinnitus severity as measured by the THI [42]. The three age categories (18–49 years, 50–69 years, ≥70 years) were informed by population statistics for moderate to severe hearing loss sourced from the Royal National Institute for Deaf People, then Action on Hearing Loss [43]. Allocation codes were created for each eligible participant after visit 1 and before visit 2.

Allocation was concealed from the researchers involved in outcome assessment and from the audiologists fitting or adjusting the device, by a device programming code. On randomisation, the audiologist was issued with a code by a team member not in contact with participants or involved in outcome assessment or analysis. This code was entered by the audiologist into the neurostimulator programme console, and this determined whether the participant was issued with a device programmed to either treatment or control. Allocation was triple-blind because participants did not know the group to which they are allocated, and the data analysts knew only whether participants were in group ‘A’ or ‘B’, not which group was treatment or control.

### 2.9. Monitoring of Data Entry

To check for accuracy in transcribing data from the paper-based case report forms to the electronic records, a process of source data verification was conducted by team members who were not involved in data analysis. For the RCT, we took a random sample of 10% of the primary time points (visits 2 and 6) for each centre and checked that outcome scores were correctly recorded. There were eight transcription errors out of 9869 data entries (0.08%), confirming high transcription accuracy. For the open-label LTE, we took a random sample of questionnaire data collected for 18 participants at visits 9 and 10 and checked that scores were correctly recorded. There were 12 transcription errors out of 1800 data entries (0.67%) confirming high transcription accuracy.

### 2.10. Statistical Methods

**Figure 2:**
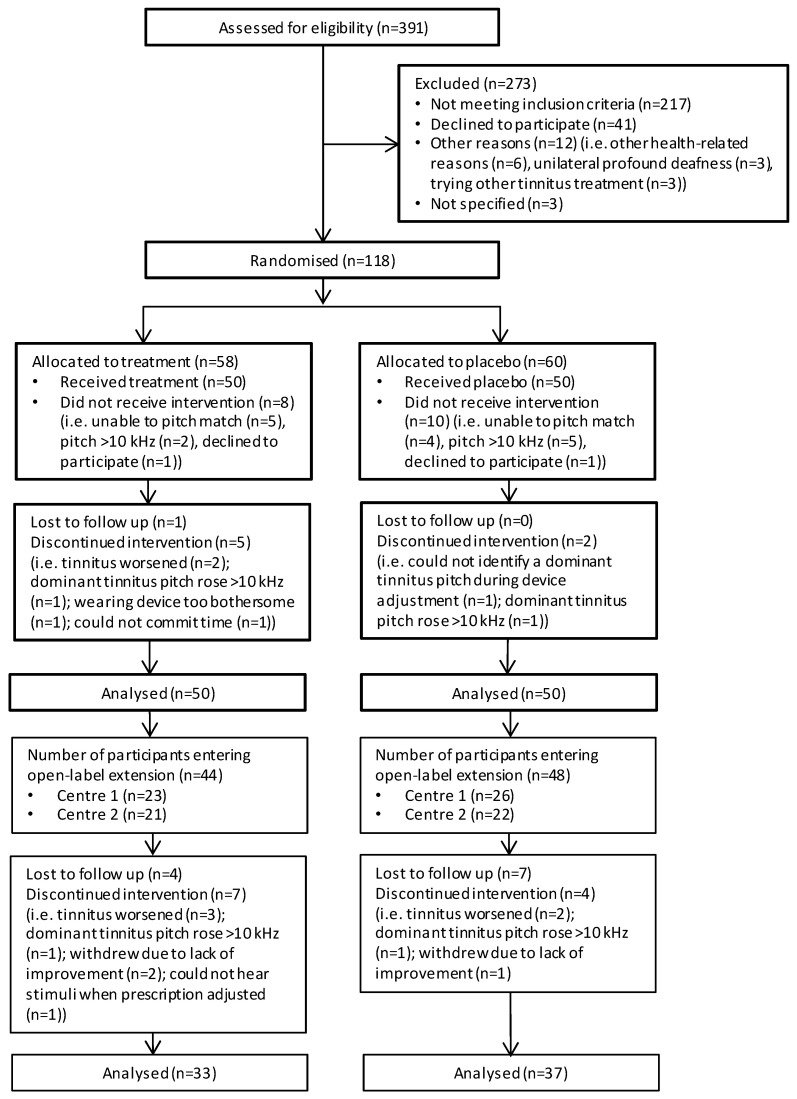
Flow chart for the RESET2 clinical trial. RCT phase is denoted by the thicker outlined boxes.

Data analyses were performed in SPSS (v21.0) [44] and R (v3.0.0) [45]. Descriptive statistics were created for primary and secondary outcomes at the eligibility assessment (visit 1) and visits 2, 4, 5, 6 and 10 using the available (i.e., non-imputed) data. An intention-to-treat analysis was used to prevent bias caused by the loss of participants (n = 8, Figure 2). Interpretation was based on analyses conducted on multiple imputed datasets using Rubin’s rule to combine estimates and estimate standard errors [45,46]. The ‘mice’ package in R was used to obtain five imputed datasets.

To assess for differences in responsiveness to intervention between the treatment and placebo groups, the dependent variables were computed by taking the difference between visits 2 and 6 (visit 6—visit 2) on each measure. Adjusted differences were tested using linear regressions of outcome on randomised group, centre, and the factors employed in minimising participants to groups, i.e., gender, age, hearing loss, and grade of tinnitus severity at the eligibility assessment (visit 1). Covariates were included in the regression models regardless of statistical significance. The imputed unadjusted differences between groups were assessed using independent-group *t*-tests and complete case analyses were also performed, for cross-validation with the imputed adjusted results.

The second endpoint and planned analysis was at the 24-week stage to examine the effect of 12 weeks’ intervention across all 100 participants. The final analysis of long-term effects was to examine all 100 participants at trial completion (placebo group = 24 weeks treatment, treatment group = 36 weeks treatment). Differences between time points were assessed using repeated measures ANOVA and complete case analyses were also performed, for exploration of the data and cross-validation with the imputed results. Bonferroni correction was applied to adjust for multiple comparisons of secondary outcomes.

Heterogeneity in the tinnitus population is well known, and between-subject variability in the response to intervention might mask individual effects that are clinically meaningful and scientifically informative [28,47]. Planned subgroup analysis used ordinal regression to investigate whether any of the eligibility assessment measures might predict a clinically meaningful response to treatment as defined by a reduction of at least 7.19 points on the THQ. Choice of predictors was informed a priori by clinical experience at The Tinnitus Clinic (Mark Williams, personal communication). Predictors were grades of tinnitus severity (‘mild/moderate/severe’) defined by the THI, tinnitus type (‘tonal/ringing/hissing’) identified using the Tinnitus Tester, self-reported tinnitus duration (in years), self-reported aetiology (‘noise exposure/ other/don’t know’) and effect on sleep (‘yes/no/don’t know’) identified by the TCHQ.

Questionnaires can have reduced responsiveness to treatment-related change when a large proportion of participants respond at baseline at either extreme of the response scale: floor effects (scores at the minimum of the response scale) reduce sensitivity to detecting group-level improvement, while ceiling effects (scores at the maximum of the response scale) reduce sensitivity to detecting group-level worsening [48]. One exploratory analysis therefore considered sensitivity of our chosen tinnitus questionnaires to detect changes from baseline in the study sample. We used the essential criteria defined by Terwee et al. [48] which states that less than 15% of respondents should achieve the lowest or highest possible score from examination of the item response distributions.

### 2.11. Fidelity of the Trial Procedures

Three minor deviations from the published protocol were notified to the Sponsor and Ethics Committee. First, the device fitting protocol specified the audiologist to operate the pitch matching controls rather than the patient, which did not fully reflect the manufacturer’s recommended fitting instructions [49]. The methods used for pitch matching and device fitting were informed by training given to the study team by the funder and manufacturer, and these were deemed to adequately reflect normal conditions of use in the clinic [49]. Second, regarding the exclusion criteria “Absolute thresholds >70 dB on individual frequencies up to 8 kHz (unable to sufficiently hear the stimulus)”, we extended this criterion to include the next frequency above that identified as the participant’s dominant tinnitus pitch (fT) because the intervention required audibility of the stimulation tones presented above the dominant tinnitus pitch. Hence, the upper limit for this exclusion criterion was 11.2 kHz, not 8 kHz. Third, we did not assess tinnitus loudness and annoyance using the VAS, with stimulation on, to maintain the integrity of the EEG measures which required a minimum 2 h break from stimulation (see also [19]). Additionally, we excluded from participation anyone who was receiving another form of treatment for tinnitus so that it would not interfere with the trial results and interpretation. This exclusion criterion was not explicitly stated in the published protocol [26] but had been defined as a criterion for withdrawal of enrolled participants. None of these factors were individually critical but have been considered in terms of how they might influence interpretation of clinical efficacy [49].

## 3. Results

### 3.1. Participant Flow

Figure 2 illustrates the flow of participants throughout the trial. Three-hundred and ninety-one individuals were screened for eligibility across the two centres before 100 eligible participants were identified and recruited into the trial. From the 391 individuals screened, 217 did not meet the study inclusion criteria. Of these, we excluded 96 participants (Nottingham = 53, London = 43) at the point of telephone screening, and 177 participants (Nottingham = 121, London = 56) at the subsequent eligibility assessment (visit 1).

Table 2 details reasons for exclusion. Forty-one individuals declined to participate and a further 15 could not participate for other reasons.

One-hundred and eighteen individuals were invited to attend for the first collection of outcome data and for their device fitting (visit 2). The total number of individuals seen at visit 2 exceeded 100 because 18 did not receive the intervention (Nottingham = 10, London = 8). Typically, they did not meet the prescription protocol for device fitting; nine were unable to complete the adaptive bracketing method for identifying the dominant tinnitus pitch, seven completed the procedure but matched their dominant tinnitus pitch to a frequency above 10 kHz, and two declined to participate in the study after successful eligibility screening. One participant was lost to follow up during the RCT and 11 during the open-label LTE.

### 3.2. Characteristics of the Eligible Participants

Demographic and clinical characteristics of the 100 participants fitted with a device are reported in Table 3. Individual and mean hearing thresholds per ear and per group are given in Supplementary Figure S1. There were no statistically significant differences between groups. Participants had an average age of 50 years, experienced moderate tinnitus symptom severity, and minimal generalised anxiety or depression. The ratio of men to women was approximately 3:1. The groups were well matched with respect to the four minimisation variables (age, gender, hearing loss, grade of tinnitus severity).

### 3.3. Adverse Events

Two participants were withdrawn from the treatment arm of the RCT within two weeks of being fitted with the device due to adverse events, i.e., our pre-defined withdrawal criteria that participants reported their tinnitus had worsened and had become unbearable. Two further participants were withdrawn during the open-label LTE for the same reason. No serious adverse events occurred during the trial.

### 3.4. Implementation of the Intervention

Five audiologists provided fitting during the RCT. All audiologists were appropriately trained, and the same fitting protocol was used throughout across both study sites. Table 4 and Table 5 highlight the fidelity of the audiology appointment dates assessed against protocol.

In addition to the five audiology visits expected in the RCT, four participants from the Nottingham centre (two treatment and two placebo) attended one additional audiology visit each and one participant from the placebo group attended two additional visits because they required recalibration of their device. Five of these additional visits took place between visit 3 and 4 (placebo group = day 32 (SD 5), treatment group = day 30 (SD 6)) and one between visit 5 and 6 (placebo group = day 78). From a maximum of 500 audiology visits during the RCT across both centres, as described by the study protocol, 465 were attended (Nottingham = 252, London = 213). Of the 35 visits not attended, 24 were because of participant withdrawal from the study (Nottingham = 19, London = 5) and 11 were missed appointments (Nottingham centre = 4, London centre = 7).

Participants were required to complete a written usage diary that included the listening situation and the number of hours for which the device was worn each day. We analysed the device-usage data of those 45 participants who showed the greatest change in THQ score (either ‘increase’ or ‘decrease’) (Nottingham = 25, London = 20). For this subset, mean daily usage was 4.79 h (SD 0.23) (Nottingham = 4.81 (SD 0.27), London = 4.77 (SD 0.32)) with no observable difference between those whose THQ score increased or decreased. Participant adherence with usage instructions was therefore acceptable and did not appear to influence change in THQ score.

During the audiology appointments, the participant would have their device programmed according to their tinnitus percept, following the device fitting protocol. Ninety-one participants had their devices programmed at each audiology visit following an assessment of the dominant tinnitus pitch of each ear with tinnitus. Nine participants at the London centre with bilateral tinnitus had their devices programmed at each of the audiology visits by an assessment of the dominant pitch in one ear followed by matching the frequency in the other ear. During the audiology visits of the RCT, two participants at the London centre were withdrawn because they were unable to hear all the prescription tones and two participants at the Nottingham centre were withdrawn due to their dominant tinnitus pitch being >10 kHz (Figure 2).

### 3.5. Fidelity of Outcome Assessments

Table 4 and Table 5 show good fidelity of research appointment intervals assessed against the protocol.

### 3.6. Planned Analyses

#### 3.6.1. Primary Analysis

Descriptive statistics are given in Table 6 for global scores on planned primary and secondary outcomes at visits 2, 6, and 10. Only completed cases are reported and so the number of participants contributing to each mean score is denoted in italics. Mean changes over time and the differences between groups were relatively small and the variance between subjects was large.

From statistical analysis of the primary outcome, adjusted (*p* = 0.85) and unadjusted (*p* = 0.90) imputations demonstrated there was no difference in the change on global THQ score between the treatment and placebo groups (Table 7). Table 7 reports all estimated mean differences between groups of the change following intervention (visit 6—visit 2) based on imputed data and adjusted on the minimisation variables (gender, age, hearing loss and tinnitus severity), on group allocation and centre. The covariates ‘centre’ and baseline tinnitus severity were carefully inspected to confirm that they had no influence on the outcome. This was true for both the global THQ, and all secondary outcomes (*p* > 0.05). Because the effects were not statistically significant, we do not report effect sizes, but instead report the 95% confidence intervals for all mean difference values. The same pattern of findings was found in the complete case analyses (adjusted *p* = 0.92; unadjusted *p* = 0.91) (Supplementary Table S1). No matter what the analysis, the mean difference in THQ between groups was less than one point on the 100-point scale. This mean change is markedly less than the clinically meaningful difference (7.19) that we estimated from previous data [39].

#### 3.6.2. Secondary Analyses

Table 8 provides descriptive statistics of the pooled groups used to evaluate within group effects at 12-, 24-, and 36-weeks treatment. Analysis of the 12-week treatment THQ data (treatment group at week 12 and placebo group at week 24) showed there to be no significant change compared to baseline on either multiple imputed (*p* = 0.34) or complete case (*p* = 0.02) data when corrected for multiple comparisons (Supplementary Table S2). For both multiple imputed and complete case data mean changes at 12 weeks ranged from −28 to +48 points. Amongst complete cases, a clinically meaningful improvement in global THQ score was observed for 20 of 84 (24%) participants after 12 weeks treatment. For nine participants (11%) THQ score was worse by >7 points after 12 weeks.

After 24-week treatment (end of trial for placebo group) there was no significant within group change compared to baseline on imputed (*p* = 0.61) or complete case data (*p* = 0.08) (Supplementary Table S3). For multiple imputed data change at 24 weeks ranged from −14 to +26, and for complete case it ranged −15 to +9. For complete case data a clinically meaningful improvement in global THQ score was observed for 5 of 35 (14%) participants after 24 weeks treatment (complete case data, placebo-crossover group). Just one participant (3%) had an increase in THQ score >7 points at 24 weeks.

After 36 weeks treatment there was no significant within group change compared to baseline for either multiple imputed (*p* = 0.30) or complete cases (*p* = 0.06) (Supplementary Table S4). For both multiple imputed and complete case data changes in THQ score after 36 weeks were highly variable ranging from −45 to +35. For complete cases a clinically meaningful improvement in global THQ score was observed for 12 of 30 (40%) participants after 36 weeks treatment. Five participants (17%) had an increase in THQ score >7 points at 36 weeks.

All other secondary outcomes (TFI, THI, and overall quality of life) remained unchanged throughout the trial period (Table 7). Perceptual measure of tinnitus as measured by the VAS and the Tinnitus Tester programme were also no different between treatment and placebo groups (Table 6). For all secondary outcomes, the complete case analyses confirmed the same interpretation of the data (Supplementary Table S1). Results of the planned subgroup analysis using ordinal regression on the global THQ showed that none of the suggested baseline factors were predictive (*p* > 0.05). Significance values for each predictor variable were as follows: tinnitus severity *p* = 0.29, tinnitus type *p* = 0.49, tinnitus duration *p* = 0.15, aetiology *p* = 0.21, and effect on sleep *p* = 0.30. This finding agrees with RESET1 where the authors found that the treatment-related change in global TQ did not depend on classification of baseline tinnitus severity as ‘more/less severely impaired’, tinnitus duration, or age [19].

### 3.7. Exploratory Analysis

For the THQ, 11 out of the 27 items reaching the criterion for floor or ceiling effects. Five items from the ‘Social, emotional and behavioural tinnitus effects’ subscale (#7, #15, #16, #19, #23) and six items from the ‘Tinnitus and Hearing’ subscale (#2, #6, #20, #21, #22, #26) were at floor for ≥15% of the sample. Ceiling effects were found on the remaining two items from the ‘Social, emotional and behavioural tinnitus effects’ subscale (#10, #22), while all items from the ‘Outlook on tinnitus’ subscale (#1, #3, #5, #8) were also at ceiling.

## 4. Discussion

In a two-centre, double-blind, placebo-controlled, parallel-group randomised trial we found no statistically or clinically meaningful difference on a battery of tests between the verum stimulation algorithm delivered by the T30 neurostimulator and a placebo algorithm. This first efficacy study therefore concludes that at a group level the verum stimulation algorithm delivered by the T30 neurostimulator is no more efficacious than an active placebo using sound, in terms of change in tinnitus symptom severity, health-related quality of life, or tinnitus perceptual characteristics. In a secondary planned analysis, we also found no significant within group effect on tinnitus symptom severity of 12-, 24-, or 36-weeks treatment.

These null findings should of course be considered in the context of challenges and progress in the design and delivery of clinical trials for tinnitus. Indeed, landmark trials in the field of tinnitus are few. Exceptions might include Cima et al. [50], who trialled CBT for tinnitus, Hall et al. [51] who trialled a novel potassium channel modulator, and Conlon et al. [52] who in the TENT A study trialled a novel bimodal stimulation intervention. Notably, in the RESET2 trial, the stimulation parameters which defined the control condition were not predicted to induce any neuroplastic reorganisation according to the manufacturer’s underlying theoretical assumptions. Hence, the term ‘control’. The hypothesis under test here was whether the verum neurostimulation was more effective than the placebo control. In the TENT A study [52], the stimulation parameters of all three experimental conditions were expected to induce a therapeutic benefit, although perhaps to a greater or lesser degree. All conditions were statistically compared to one another without there being any single ‘control’ condition. The premise of the study was to explore which parameter settings would be most effective for bimodal neuromodulation.

Whatever the intervention, trials in tinnitus are complicated by the lack of objective outcome measures, lack of clarity in what is the minimal important change in a patient-reported outcome measure, and the heterogeneity of tinnitus in terms of perceptual characteristics, symptom severity, comorbidities, personal characteristics, etc. The current trial relied on the THQ as the primary outcome measure. Responsiveness to treatment-related change may have been partially compromised by the reduced sensitivity of the THQ due to floor and ceiling effects on several items. One might argue that the VAS measures of loudness and annoyance and the three perceptual measures of tinnitus should mitigate against the limitations of questionnaire measures since other evaluations of Acoustic CR^®^ Neuromodulation therapy have reported positive findings on some of these variables [19,22,23]. However, we observed no significant change over time between treatment and placebo groups on any of these secondary measures. Finally, on the issue of outcome measures, we only recently have an established core outcome set (COS) that have been agreed by international consensus as critical to be measured in all trials of interventions for tinnitus [53]. The COS for sound-based interventions comprises “intrusiveness”, “ability to ignore,” “concentration,” “quality of sleep,” and “sense of control”, which if used as outcomes in future trial and measured using well-validated tools, will likely identify even small but important treatment-related changes and between group difference, and allow for greater comparison of interventions and synthesis of trial findings.

Another variable to consider in interpreting our results is the timing of the outcome measurement. It is reasonable to question whether 12-weeks of device usage is sufficient for significant differences in between-group benefit to accrue. This was the timescale used in the design of RESET1, and at the time of delivering this trial was the only evidence on which to base our design. We note that subsequent uncontrolled studies reported alleviation of tinnitus after 6 and 12 months of Acoustic CR^®^ Neuromodulation treatment [22,23]. Our trial involved an uncontrolled and unblinded open-label extension and measurement of tinnitus symptom severity for up to 36 weeks of treatment. We observed no significant benefit in this small group permitting only speculation on the true long-term potential for benefit of this treatment approach.

Choice of treatment algorithm is also a consideration. Our study-specific pitch matching procedure differed slightly from manufacturer’s recommendations [49] but there does appear to be some acceptable degree of tolerance concerning the stimulation frequencies of the verum stimulation algorithm. For example, Hauptmann et al. [23] and Theodoroff et al. [24] placed stimulation tones at a “frequency range: 0.76 fT: 1.4 fT” (pp. 2) which differed from the therapeutic signal (frequency range: 0.5 fT: 2.0 fT) preferred in RESET1 [19] and implemented in clinical practice according to a subsequent observational study [22]. Hauptman et al. [23] suggested that pitch matching should be within +/−5% of the true tinnitus pitch for optimal therapeutic desynchronisation. That study acknowledged the variability inherent in “a traditional manual pitch-matching method” (pp. 1) and has developed an automated adaptive pitch-matching method which reduces the deviance of matching to an external tone, compared to the manual method. The authors concluded that this new approach “offers several features that are of importance for clinical trials … [and is]… expected to improve the quality of measures in tinnitus therapy effectiveness” (p. 9).

RESET2 was not a test of the effectiveness of Acoustic CR^®^ Neuromodulation treatment as delivered in normal clinical practice. In designing this trial, we sought to address previous criticism in the field that trials of sound therapy for tinnitus have been unable to separate the acoustic component of audiological treatment from the psychological component of counselling. Hence, RESET2 was designed specifically to test the efficacy of the verum stimulation algorithm delivered by the T30 neurostimulator against a placebo sound sequence delivered by the same device. It was carried out in a controlled experimental setting that carefully constrained the interactions between research staff and participants to isolate the acoustic component of the therapy. An inherent part of routine audiological practice involves identifiable professional-client interactions which are not specific to the acoustic treatment per se, but nevertheless could contribute therapeutic ingredients of Acoustic CR^®^ Neuromodulation treatment. Examples of such ingredients include creation of a positive therapeutic alliance, education and reassurance about tinnitus, and application of psychologically informed therapeutic techniques. Certainly, these are known to be common in many tinnitus therapies [54]. In order not to contaminate data interpretation, our trial protocol and staff training procedures did not extend to these behaviours, nor was time allocated for anything more than the trial assessments and device programming. One might normally expect unspecific effects to apply to all participants and so it is particularly interesting that our findings showed no change from baseline even in the placebo group. This null finding could be indicative that our intervention procedures successfully avoided these unspecific therapeutic effects. Another aspect of unspecific positive effect that must be considered is in the sham/placebo control sound stimulation. Here we used what was considered the optimal placebo that would mimic the acoustic sensation of the intervention without inducing the therapeutic desynchronisation of cortical activity. However, this was still an active condition using sound across a frequency range in what might be considered a therapeutic manner. Factors such as measurement error and tolerance levels for change in tinnitus pitch over time also need to be carefully considered [49].

We followed strict inclusion criteria during participant recruitment that were based on the manufacturer’s suitability criteria for the Acoustic CR^®^ Neuromodulation treatment. Additional criteria were also used to account for potential confounding factors, such as the use of other tinnitus treatments or being fitted with hearing aids less than nine months previously. About 55% (n = 217) of respondents assessed for eligibility (n = 391, Figure 2) were excluded based on these criteria. Employing these criteria together with minimisation variables was advantageous to the homogeneity of the intervention groups with respect to tinnitus and hearing characteristics. However, it should be borne in mind that our sample does not represent chronic tinnitus in the general population, nor in a typical clinical sample.

## 5. Conclusions

From the results of this first RCT of Acoustic CR^®^ Neuromodulation treatment, there is no evidence to conclude that the medical device provides significant therapeutic benefit to patients. Although this study design compared the treatment to a well-matched placebo sound intervention, we hypothesise that Acoustic CR^®^ Neuromodulation is unlikely to demonstrate clinical superiority over other sound-based tinnitus interventions or, given its recent comparison with a short-form CBT, superiority over psychological interventions. Further trials would be required to inform commercialisation decisions for this treatment, e.g., evidence of effectiveness in clinical trials required to gain Food and Drug Administration approval for the US market. This contrasts with European CE marking for medical devices, which only requires a post-market clinical follow-up study once the CE Mark is obtained. Should further trials be conducted to investigate the clinical effectiveness of this medical device, then we would propose that alternatives to the conventional RCT design are considered. Comparisons with other tinnitus treatments may be of interest to inform shared clinical decision making and to expand patient choice. Within-subject comparisons may also be more informative than group mean comparisons but would require this built into the trial design at the outset.

## Figures and Tables

**Table 1:** Inclusion and exclusion criteria assessed during the telephone screening and eligibility assessment (visit 1).

Inclusion Criteria
Men and women ≥ 18 years of age
Pure-tone average (PTA) hearing thresholds < 60 dB HL (0.5, 1, 2, 4 kHz)
Must be able to hear stimulation tones presented by the device at all frequencies
Chronic subjective tinnitus for more than 3 months
Dominant tinnitus pitch corresponding to a frequency between 0.2 and 10 kHz (practical lower boundary was 0.5 kHz due to test limitations with the Tinnitus Tester)
At least mild tinnitus, score ≥ 18 measured on Tinnitus Handicap Inventory (THI)
Willing to wear the device for 4 to 6 h daily during the trial
Sufficient command of English language to read, understand and complete the questionnaires
Able and willing to give informed consent
Exclusion criteria
Objective tinnitus, Ménière’s disease, temporomandibular joint disorder
Acoustic Neuroma
Pulsatile tinnitus
Intermittent tinnitus
Severe anxiety, >25 score measured on the Beck Anxiety Inventory (BAI)
Severe depression, >29 score measured on the Beck Depression Inventory (BDI-II)
Catastrophic tinnitus, score ≥ 78 measured on the Tinnitus Handicap Inventory (THI)
Hearing aid wearer for less than 9 months, or long-term hearing aid wearer has had a prescription adjustment within the last 3 months
Pure-tone absolute hearing thresholds > 70 dB on individual frequencies up to 11.2 kHz (unable to sufficiently hear the stimulus)
Taking part in another trial during the 30 days before study start
The individually tailored training stimulus is uncomfortable or not acceptable to the participantCurrently receiving another form of treatment for tinnitus

**Table 2:** Breakdown of the reasons for excluding 217 participants (specific criteria listed in the protocol).

Exclusion Reason	Total
Ménière’s disease	6
Acoustic neuroma	2
Temporo-mandibular joint disorder	3
Pulsatile tinnitus	7
Somatosensory modulated tinnitus	17
Intermittent tinnitus	7
Slight tinnitus (THI score < 18)	35
Catastrophic tinnitus (THI score ≥ 78)	6
Dominant tinnitus pitch <0.2 kHz or >10 kHz	56
Could not identify a dominant tinnitus pitch	7
Hearing loss > 70 dB HL at next frequency above dominant tinnitus pitch or unable to hear intervention sounds	47
Pure-tone audiometric average > 60 dB HL (0.5, 1, 2, 4 kHz) in tinnitus ear	2
First-time hearing-aid user (<9 months)	11
Long-term hearing-aid user, but had recent fitting adjustments within last 3 months	2
Severe anxiety (BAI score > 25)	4
Severe depression (BDI score > 29)	5

**Table 3:** Participant characteristics at the eligibility assessment (visit 1). SD = standard deviation.

	Treatment (Mean, SD)	Placebo (Mean, SD)	Difference between Groups
Age	49.1 (11.3) years	51.8 (12.2) years	*t* = 1.15, *p* = 0.25
Gender	33 male: 17 female	37 male: 13 female	*X*^2^ = 0.76, *p* = 0.38
Hearing level (pure tone average) dB HL	15.85 (10.86)	16.45 (11.96)	*t* = −0.26, *p* = 0.79
Tinnitus Handicap Inventory (THI)	40.7 (16.4)	41.4 (16.0)	*t* = 0.18, *p* = 0.89
Beck Anxiety Inventory (BAI)	4.7 (4.7)	5.4 (6.6)	*t* = 0.67, *p* = 0.51
Beck Depression Inventory (BDI)	8.4 (6.0)	9.9 (7.3)	*t* = 0.47, *p* = 0.32

**Table 4:** Fidelity of audiology and research appointments in the RCT. Audiology appointments were for clinical assessment and follow-up. Research appointments were restricted to outcome assessments. Fidelity is calculated relative to the initial device fitting (visit 2), with visit 6 at 12 weeks after initial device fitting. Mean across participants in days; standard deviation in brackets.

Visit Number	1	2	3	4	5	6
Day as per protocol	N/A	t0	14	28	56	84
Audiology appointments						
Treatment	N/A	t0	16(3)	32 (6)	61 (8)	89 (7)
Placebo	N/A	t0	17(4)	32 (5)	58 (6)	87 (7)
Research appointments						
Treatment	−40 (35)	t0	N/A	32 (6)	61 (8)	89 (7)
Placebo	−41 (32)	t0	N/A	32 (5)	58 (6)	87 (7)

**Table 5:** Fidelity of audiology and research appointments in the open-label extension. Audiology appointments were for clinical assessment and follow-up. Research appointments were restricted to outcome assessments. Fidelity is calculated relative to the end of the RCT phase (visit 6), with visit 10 at 36 weeks after initial device fitting. Mean across participants in days; standard deviation in brackets.

Visit Number	6	7	8	9	10
Day as per protocol	t0	14	42	84	168
Audiology appointments					
Treatment	t0	NA	44 (13)	86 (12)	167 (15)
Placebo	t0	15 (7)	44 (10)	87 (10)	173 (11)
Research appointments					
Pooled	t0	NA	NA	87 (11)	170 (13)

**Table 6:** Descriptive statistics for the primary outcome measure (THQ) and secondary outcome measures (WHOQOL-BREF, THI, TFI, loudness, annoyance, pitch and bandwidth), for complete cases only. SD = standard deviation; VAS = visual analogue scale.

	Treatment (Mean, SD)		Placebo (Mean, SD)		All Participants (Mean, SD)
	Visit 2	Visit 6	Visit 2	Visit 6	Visit 10
THQ	n = 48	n = 44	n = 45	n = 48	n = 67
Global score	44.05 (16.70)	40.87 (18.30)	46.16 (15.97)	42.52 (16.86)	41.25 (19.15)
WHOQOL-BREF	n = 49	n = 44	n = 46	n = 48	
Quality of life (Q1)	3.86 (0.84)	4.05 (0.86)	3.89 (0.67)	4.00 (0.90)	Not measured
THI	n = 49	n = 44	n = 46	n = 48	
Global score	36.86 (19.38)	34.50 (19.97)	37.96 (18.57)	36.75 (20.49)	Not measured
TFI	n = 49	n = 44	n = 46	n = 48	n = 69
Global score	42.33 (18.75)	40.80 (21.83)	43.64 (20.39)	39.72 (20.98)	39.53 (22.40)
VAS loudness (Q2)	6.31 (2.03)	5.86 (2.30)	6.65 (1.78)	6.00 (2.10)	5.93 (2.24)
VAS annoyance (Q3)	3.80 (2.69)	3.75 (2.71)	4.33 (2.92)	3.63 (2.73)	3.87 (2.77)
Tinnitus Tester	n = 46	n = 44	n = 47	n = 47	
Loudness (dB)	15.24 (16.53)	16.60 (20.49)	19.14 (15.52)	19.81 (17.00)	Not measured
Pitch (kHz)	6.76 (3.43)	6.60 (2.77)	7.72 (3.12)	8.00 (3.30)	Not measured
Bandwidth (kHz)	3.02 (0.76)	2.99 (0.66)	2.92 (0.72)	2.92 (0.64)	Not measured

**Table 7:** Estimated mean differences between groups in terms of responsiveness to intervention using imputed data (visit 6—visit 2). Adjustment for randomised group, trial centre, and the factors employed in minimising participants to groups (i.e., gender, age, hearing loss and THI at the eligibility assessment) (visit 1). Tinnitus loudness in dB; Tinnitus pitch and bandwidth in kHz.

	Imputed Data with Adjustment			Imputed Data without Adjustment		
	Mean Difference	*p* Value	95% Confidence Interval	Mean Difference	*p* Value	95% Confidence Interval
RCT analysis at 12 weeks						
Global THQ	−0.45	0.85	−5.25 to 4.35	−0.30	0.90	−5.11 to 4.52
WHOQOL-BREF	−0.05	0.80	−0.40 to 0.31	−0.03	0.86	−0.40 to 0.34
Global THI	0.69	0.79	−4.49 to 5.86	1.09	0.68	−4.13 to 6.31
Global TFI	−2.59	0.48	−9.77 to 4.60	−2.14	0.42	−7.39 to 3.11
VAS loudness (Q2)	−0.08	0.89	−1.18 to 1.03	−0.02	0.98	−1.28 to 0.08
VAS annoyance (Q3)	−0.92	0.21	−2.36 to 0.52	−0.93	0.19	−2.35 to 0.48
Tinnitus loudness	−1.49	0.68	−8.87 to 5.89	−1.76	0.63	−9.28 to 5.76
Tinnitus pitch	0.12	0.87	−1.39 to 1.73	0.22	0.77	−1.28 to 1.72
Tinnitus bandwidth	0.03	0.84	−0.31 to 0.38	0.03	0.87	−0.32 to 0.38
Open-label extension at 36 weeks						
Global THQ	−2.34	0.30	−6.73 to 2.05	−5.41	0.06	−11.00 to 0.19
Global TFI	−0.48	0.88	−6.60 to 5.64	−8.91	0.04	−15.89 to −0.48

**Table 8:** Descriptive statistics for the primary (THQ) and secondary (TFI) outcome measures used in within-group comparisons.

	Baseline	12 Weeks Treatment	24 Weeks Treatment	36 Weeks Treatment
THQ	n = 95	n = 86	n = 35	n = 33
Global score	43.28 (16.71)	41.59 (17.93)	41.82 (18.21)	40.61 (20.39)
Soc, emot & beh	41.42 (20.65)	38.84 (21.22)	38.76 (21.44)	38.76 (24.09)
Hearing	43.07 (23.23)	41.87 (22.43)	42.17 (24.84)	41.28 (23.41)
TFI				
Global score	41.55 (19.69)	39.42 (21.64)	39.63 (21.54)	39.23 (23.94)
Intrusive	52.82 (22.07)	49.42 (23.46)	54.29 (22.13)	47.88 (23.67)
Sense of control	50.03 (22.44)	47.36 (25.28)	50.28 (26.18)	47.17 (26.16)
Cognitive	37.46 (24.84)	35.52 (26.36)	33.24 (27.18)	37.87 (29.14)
Sleep	41.24 (31.30)	40.92 (32.21)	34.44 (29.06)	42.73 (35.18)
Auditory	36.42 (28.24)	35.40 (28.14)	35.83 (29.40)	36.16 (23.95)
Relaxation	54.26 (27.92)	51.34 (28.72)	53.43 (27.32)	49.09 (30.42)
Quality of life	29.09 (24.12)	28.82 (25.18)	28.68 (24.33)	28.18 (26.80)
Emotional	35.15 (27.18)	30.19 (27.10)	31.57 (26.47)	28.48 (28.27)

THQ = Tinnitus Handicap Questionnaire; TFI = Tinnitus Functional Index; Soc, emot and beh = social, emotional and behavioural effects subscale; SD = standard deviation.

## Data Availability

Data is freely available on request to the authors.

## Supplementary Materials

The following supporting information can be downloaded at: https://www.mdpi.com/article/10.3390/brainsci12030317/s1, Table S1: Estimated mean differences between groups in terms of responsiveness to intervention using complete cases only (visit 6—visit 2); Table S2: Estimated mean differences within whole group in terms of responsiveness to intervention using imputed and complete case data (12 weeks); Table S3: Estimated mean differences within group in terms of responsiveness to intervention using imputed and complete case data (24 weeks); Table S4: Estimated mean differences within group in terms of responsiveness to intervention using imputed and complete case data (36 weeks). Figure S1: Individual and average pure tone thresholds per ear per group.

## References

[1] McCormack A., Edmondson-Jones M., Somerset S., Hall D. (2016). A systematic review of the reporting of tinnitus prevalence and severity. Hear. Res..

[2] Rosing S.N., Schmidt J.H., Wedderkopp N., Baguley D.M. (2016). Prevalence of tinnitus and hyperacusis in children and adolescents: A systematic review. BMJ Open.

[3] De Ridder D., Schlee W., Vanneste S., Londero A., Weisz N., Kleinjung T., Shekhawat G.S., Elgoyhen A.B., Song J., Andersson G. (2021). Tinnitus and tinnitus disorder: Theoretical and operational definitions (an international multidisciplinary proposal). Progress in Brain Research.

[4] Watts E.J., Fackrell K., Smith S., Sheldrake J., Haider H., Hoare D.J. (2018). Why is tinnitus a problem? A qualitative analysis of problems reported by tinnitus patients. Trends Hear..

[5] Sereda M., Xia J., El Refaie A., Hall D.A., Hoare D.J. (2018). Sound therapy (using amplification devices and/or sound generators) for tinnitus. Cochrane Database Syst. Rev..

[6] Fuller T., Cima R., Langguth B., Mazurek B., Vlaeyen J.W., Hoare D.J. (2020). Cognitive behavioural therapy for tinnitus. Cochrane Database Syst. Rev..

[7] Nondahl D.M., Cruickshanks K.J., Huang G.H., Klein B.E.K., Klein R., Javier Nieto F., Tweed T.S. (2011). Tinnitus and its risk factors in the Beaver Dam Offspring Study. Int. J. Audiol..

[8] Gopinath B., McMahon C.M., Rochtchina E., Karpa M.J., Mitchell P. (2010). Risk factors and impacts of incident tinnitus in older adults. Ann. Epidemiol..

[9] Baguley D., McFerran D., Hall D. (2013). Tinnitus. Lancet.

[10] Eggermont J.J. (2005). Tinnitus: Neurobiological substrates. Drug Discov. Today.

[11] Schaette R., Kempter R. (2009). Predicting tinnitus pitch from patients’ audiograms with a computational model for the development of neuronal hyperactivity. J. Neurophysiol..

[12] Schaette R., McAlpine D. (2011). Tinnitus with a normal audiogram: Physiological evidence for hidden hearing loss and computational model. J. Neurosci..

[13] Roberts L.E. (2011). Neural Synchrony and Neural Plasticity in Tinnitus. Textbook of Tinnitus.

[14] Tass P.A., Popovych O.V. (2012). Unlearning tinnitus-related cerebral synchrony with acoustic coordinated reset stimulation: Theoretical concept and modelling. Biol. Cybern..

[15] Tass P.A. (2003). A model of desynchronizing deep brain stimulation with a demand-controlled coordinated reset of neural subpopulations. Biol. Cybern..

[16] Lysyansky B., Popovych O.V., Tass P.A. (2011). Desynchronizing anti-resonance effect of m: N ON-OFF coordinated reset stimulation. J. Neural Eng..

[17] Tass P.A., Majtanik M. (2006). Long-term anti-kindling effects of desynchronizing brain stimulation: A theoretical study. Biol. Cybern..

[18] Tass P.A., Qin L., Hauptmann C., Dovero S., Bezard E., Boraud T., Meissner W.G. (2012). Coordinated reset has sustained aftereffects in Parkinsonian monkeys. Ann. Neurol..

[19] Tass P.A., Adamchic I., Freund H.J., von Stackelberg T., Hauptmann C. (2012). Counteracting tinnitus by acoustic coordinated reset neuromodulation. Restor. Neurol. Neurosci..

[20] Goebel G., Hiller W. (1994). The tinnitus questionnaire. A standard instrument for grading the degree of tinnitus. Results of a multicenter study with the tinnitus questionnaire. HNO.

[21] Wegger M., Ovesen T., Larsen D.G. (2017). Acoustic coordinated reset neuromodulation: A systematic review of a novel therapy for tinnitus. Front. Neurol..

[22] Williams M., Hauptmann C., Patel N. (2015). Acoustic CR neuromodulation therapy for subjective tonal tinnitus: A review of clinical outcomes in an independent audiology practice setting. Front. Neurol..

[23] Hauptmann C., Ströbel A., Williams M., Patel N., Wurzer H., von Stackelberg T., Brinkmann U., Langguth B., Tass P.A. (2015). Acoustic Coordinated Reset Neuromodulation in a real-life patient population with chronic tonal tinnitus. BioMed Res. Int..

[24] Theodoroff S.M., McMillan G.P., Schmidt C.J., Dann S.M., Hauptmann C., Goodworth M.C., Leibowitz R.Q., Random C., Henry J.A. (2021). Randomised controlled trial of interventions for bothersome tinnitus: DesyncraTM versus cognitive behavioural therapy. Int. J. Audiol..

[25] Boutron I., Moher D., Altman D.G., Schulz K.F., Ravaud P. (2008). Extending the CONSORT statement to randomized trials of nonpharmacologic treatment: Explanation and elaboration. Ann. Intern. Med..

[26] Hoare D.J., Pierzycki R.H., Thomas H., McAlpine D., Hall D.A. (2013). Evaluation of the acoustic coordinated reset (CR®) neuromodulation therapy for tinnitus: Study protocol for a double-blind randomized placebo-controlled trial. Trials.

[27] Langguth B., Goodey R., Azevedo A., Bjorne A., Cacace A., Crocetti A., del Bo L., de Ridder D., Diges I., Elbert T. (2007). Consensus for tinnitus patient assessment and treatment outcome measurement: Tinnitus Research Initiative meeting, Regensburg, July 2006. Prog. Brain Res..

[28] Newman C.W., Jacobson G.P., Spitzer J.B. (1996). Development of the Tinnitus Handicap Inventory. Arch. Otolaryngol. Head Neck Surg..

[29] Roberts L.E., Moffat G., Baumann M., Ward L.M., Bosnyak D.J. (2008). Residual inhibition functions overlap tinnitus spectra and the region of auditory threshold shift. JARO.

[30] Roberts L.E., Moffat G., Bosnyak D.J. (2006). Residual inhibition functions in relation to tinnitus spectra and auditory threshold shift. Acta Oto-Laryngol..

[31] Beck A.T., Steer R.A., Ball R., Ciervo C.A., Kabat M. (1997). Use of the Beck Anxiety and Depression Inventories for Primary Care with medical outpatients. Assessment.

[32] Kuk F.K., Tyler R.S., Russell D., Jordan H. (1990). The psychometric properties of a tinnitus handicap questionnaire. Ear Hear..

[33] Fackrell K., Hall D.A., Barry J., Hoare D.J., Signorelli F., Turjman F. (2014). Tools for Tinnitus Measurement: Development and Validity of Questionnaires to Assess Handicap and Treatment Effects. Tinnitus: Causes, Treatment and Short & Long-Term Health Effects.

[34] Pierzycki R.H., McNamara A.J., Hoare D.J., Hall D.A. (2016). Whole scalp resting state EEG of oscillatory brain activity shows no parametric relationship with psychoacoustic and psychosocial assessment of tinnitus: A repeated measures study. Hear. Res..

[35] Meikle M.B., Henry J.A., Griest S.E., Stewart B.J., Abrams H.B., McArdle R., Myers P.J., Newman C.W., Sandridge S., Turk D.C. (2012). The Tinnitus Functional Index: Development of a new clinical measure for chronic, intrusive tinnitus. Ear Hear..

[36] Hoare D.J., Gander P.E., Collins L., Smith S., Hall D.A. (2012). Management of tinnitus in English NHS audiology departments: An evaluation of current practice. J. Eval. Clin. Pract..

[37] THE WHOQOL GROUP (1998). Development of the World Health Organization WHOQOL-BREF quality of life assessment. Psychol. Med..

[38] Sereda M., Hall D.A., Bosnyak D.J., Edmondson-Jones M., Roberts L.E., Adjamian P., Palmer A.R. (2011). Re-examining the relationship between audiometric profile and tinnitus pitch. Int. J. Audiol..

[39] Henry J.A., Schechter M.A., Zaugg T.L., Griest S., Jastreboff P.J., Vernon J.A., Laelin C., Meikle M.B., Stewart B.J. (2006). Clinical trial to compare tinnitus masking and tinnitus retraining therapy. Acta Oto-Laryngol. Supp..

[40] R Core Team (2013). R: A Language and Environment for Statistical Computing 2013.

[41] Altman D.G., Bland J.M. (2005). Treatment allocation by minimisation. BMJ.

[42] McCombe A., Baguley D., Coles R., McKenna L., McKinney C., Windle-Taylor P. (2001). Guidelines for the grading of tinnitus severity: The results of a working group commissioned by the British Association of Otolaryngologists, Head and Neck Surgeons, 1999. Clin. Otolaryngol..

[43] Action on Hearing Loss. https://rnid.org.uk/wp-content/uploads/2020/05/Hearing-Matters-Report.pdf.

[44] IBM Corp (2012). IBM SPSS Statistics for Windows.

[45] van Buuren S., Groothuis-Oudshoorn K. (2011). Mice: Multivariate Imputation by Chained Equations in R. J. Stat. Soware.

[46] Rubin D.B. (1987). Multiple Imputation for Nonresponse in Surveys.

[47] Tyler R., Coelho C., Tao P., Ji H., Noble W., Gehringer A., Gogel S. (2008). Identifying tinnitus subgroups with cluster analysis. Am. J. Audiol..

[48] Terwee C.B., Bot S.D.M., de Boer M.R., van der Windt D.A.W.M., Knol D.L., Dekker J., Bouter L.M., de Wet H.C.W. (2007). Quality criteria were proposed for measurement properties of health stats questionnaires. J. Clin. Epidemiol..

[49] Hall D.A., Pierzycki R.H., Thomas H., Hoare D.J. (2016). Designing and conducting a double-blind randomized placebo-controlled trial of a novel sound therapy for tinnitus: A commentary on medical device trials in ENT and Audiology. Ann. Otolaryngol. Rhinol..

[50] Cima R.F., Maes I.H., Joore M.A., Scheyen D.J., El Refaie A., Baguley D.M., Anteunis L.J.C., Breukelen G.J.P., Vlaeyen J.W. (2012). Specialised treatment based on cognitive behaviour therapy versus usual care for tinnitus: A randomised controlled trial. Lancet.

[51] Hall D.A., Ray J., Watson J., Sharman A., Hutchison J., Harris P., Daniel M., Millar B., Large C.H. (2019). A balanced randomised placebo controlled blinded phase IIa multi-centre study to investigate the efficacy and safety of AUT00063 versus placebo in subjective tinnitus: The QUIET-1 trial. Hear. Res..

[52] Conlon B., Langguth B., Hamilton C., Hughes S., Meade E., Connor C.O., Schecklmann M., Hall D.A., Leong S.L. (2020). Bimodal neuromodulation combining sound and tongue stimulation reduces tinnitus symptoms in a large randomized clinical study. Sci. Transl. Med..

[53] Hall D.A., Smith H., Hibbert A., Colley V., Haider H.F., Horobin A., Core Outcome Measures in Tinnitus (COMiT) initiative (2018). The COMiT’ID study: Developing core outcome domains sets for clinical trials of sound-, psychology-, and pharmacology-based interventions for chronic subjective tinnitus in adults. Trends Hear..

[54] Thompson D.M., Hall D.A., Walker D.M., Hoare D.J. (2017). Psychological therapy for people with tinnitus: A scoping review of treatment components. Ear Hear..

