# Supplementary material for: Systematic Evaluation of the T30 Neurostimulator Treatment for Tinnitus: A Double-Blind Randomised Placebo-Controlled Trial with Open-Label Extension
Source: Brain Sci. 2022 Feb 26;12(3):317. doi: 10.3390/brainsci12030317 (PMC8946033; doi:10.3390/brainsci12030317)
Supplement: Supplementary file 1 [file brainsci-12-00317-s001.zip › brainsci-1601157-supplementary.pdf]

brain sciences

MDPI

SUPPLEMENTARY TABLES

Supplementary Table S1. Estimated mean differences between groups in terms of responsiveness to intervention using complete cases only (visit 6 – visit 2). Adjustment for randomised group, trial centre, and the factors employed in minimising participants to groups (i.e., gender, age, hearing loss and THI at the eligibility assessment) (visit 1). Tinnitus loudness in dB; Tinnitus pitch and bandwidth in kHz.

	**Imputed data with adjustment**			**Imputed data without adjustment**		
	Mean difference	p value	95% confidence interval	Mean difference	p value	95% confidence interval
**Global THQ**	-0.25	0.92	-5.30 to 4.80	-0.27	0.91	-5.22 to 4.68
**WHOQOL-BREF**	-0.12	0.40	-0.39 to 0.16	-0.10	0.46	-0.36 to 0.17
**Global THI**	1.15	0.68	-4.31 to 6.60	1.70	0.54	-3.84 to 7.24
**Global TFI**	-2.47	0.52	-10.0 to 5.05	-2.22	0.56	-9.76 to 5.33
**VAS loudness**	-0.22	0.63	-1.11 to 0.68	-0.13	0.77	-1.02 to 0.76
**VAS annoyance**	-0.58	0.33	-1.75 to 0.60	-0.59	0.31	-1.74 to 0.56
**Tinnitus loudness**	0.62	0.81	-4.48 to 5.73	0.17	0.95	-5.39 to 5.72
**Tinnitus pitch**	0.64	0.34	-0.68 to 1.97	0.64	0.33	-0.67 to 1.96
**Tinnitus bandwidth**	0.07	0.63	-0.20 to 0.34	0.06	0.68	-0.21 to 0.32

Supplementary Table S2. Estimated mean differences within whole group in terms of responsiveness to intervention using imputed and complete case data. Comparison based on 12-week treatment – baseline.

	**Imputed data (n = 100)**			**Complete case data (n = 84)**		
	**Mean difference**	***p* value**	**95% confidence interval**	**Mean difference**	***p* value**	**95% confidence interval**
**Global THQ**	-1.06	0.34	-3.25 to 1.13	-2.58	**0.02**	-4.84 to -0.31
Soc, emot & beh	-1.42	0.32	-4.25 to 1.38	-3.13	**0.03**	-6.02 to -0.23
Hearing	-0.05	0.97	-2.64 to 2.54	-1.81	0.13	-4.19 to 0.56
**Global TFI**	-0.82	0.61	-3.97 to 2.34	-2.12	0.25	-5.77 to 1.53
Intrusive	-3.31	0.09	-7.12 to 0.51	-4.12	0.05	-8.30 to 0.07
Sense of control	-3.25	0.13	-7.42 to 0.93	-2.35	0.33	-7.07 to 2.38
Cognitive	-0.83	0.67	-4.62 to 2.97	-2.22	0.30	-6.46 to 2.01
Sleep	0.74	0.77	-4.23 to 5.71	-1.19	0.68	-6.89 to 4.50
Auditory	0.38	0.83	-3.16 to 3.92	-0.91	0.57	-6.89 to 4.50
Relaxation	-3.00	0.22	-7.79 to 1.79	-3.95	0.14	-9.29 to 1.39
Quality of life	1.46	0.48	-2.60 to 5.52	-0.59	0.79	-4.97 to 3.80
Emotional	0.50	0.83	-3.97 to 4.97	02.14	0.37	-6.85 to 2.57

THQ = Tinnitus Handicap Questionnaire; TFI = Tinnitus Functional Index; Soc, emot & beh = social, emotional and behavioural effects subscale; SD = Standard Deviation; Grey shading = significant (uncorrected); * = significant after Bonferroni correction.

Supplementary Table S3. Estimated mean differences within group in terms of responsiveness to intervention using imputed and complete case data. Comparison based on 24-week treatment – baseline.

	**Imputed data (n = 50)**			**Complete case data (n = 35)**		
	**Mean difference**	***p* value**	**95% confidence interval**	**Mean difference**	***p* value**	**95% confidence interval**
**Global THQ**	0.65	0.61	-1.90 to 3.20	-1.75	0.08	-3.73 to 0.23
Soc, emot & beh	0.32	0.84	-2.77 to 3.40	-1.70	0.17	-4.15 to 0.75
Hearing	2.26	0.23	-1.50 to 6.03	-2.24	0.14	-5.26 to 0.77
**Global TFI**	1.31	0.48	-2.36 to 4.97	-0.62	0.73	-4.26 to 3.01
Intrusive	0.20	0.93	-4.43 to 4.83	0.10	0.97	-5.16 to 5.35
Sense of control	-0.04	0.99	-5.78 to 5.70	-0.20	0.95	-6.24 to 5.85
Cognitive	1.53	0.56	-3.71 to 6.78	-0.78	0.76	-5.96 to 4.39
Sleep	-3.68	0.31	-10.81 to 3.45	-7.35	**0.03**	-14.24 to -0.47
Auditory	2.77	0.25	-2.02 to 7.57	-0.69	0.69	-4.12 to 2.75
Relaxation	0.77	0.77	-4.56 to 6.11	1.86	0.48	-3.14 to 7.13
Quality of life	4.37	0.09	-0.77 to 9.51	0.74	0.78	-4.56 to 6.03
Emotional	3.52	0.25	-2.47 to 9.51	0.88	0.79	-5.67 to 7.44

THQ = Tinnitus Handicap Questionnaire; TFI = Tinnitus Functional Index; Soc, emot & beh = social, emotional and behavioural effects subscale; SD = Standard Deviation; Grey shading = significant (uncorrected); * = significant after Bonferroni correction.

Supplementary table S4. Estimated mean differences within group in terms of responsiveness to intervention using imputed and complete case data. Comparison based on 36-week treatment – baseline.

	Imputed data (n = 50)			Complete case (n = 30)		
	**Mean difference**	***p* value**	**95% confidence interval**	**Mean difference**	***p* value**	**95% confidence interval**
**Global THQ**	-2.34	0.30	-6.73 to 2.05	-5.41	0.06	-11.00 to 0.19
Soc, emot & beh	-2.27	0.40	-7.87 to 3.14	-6.35	0.08	-13.45 to 0.85
Hearing	-1.04	0.65	-5.63 to 3.55	-4.20	0.08	-8.98 to 0.58
**Global TFI**	-0.48	0.88	-6.60 to 5.64	-8.91	**0.04**	-15.89 to -0.48
Intrusive	-1.43	0.70	-8.71 to 5.85	-10.78	**0.01**	-18.68 to -2.87
Sense of control	-1.63	0.65	-8.61 to 5.35	-6.22	0.23	-16.16 to 3.72
Cognitive	-1.16	0.76	-8.49 to 6.17	-9.00	0.06	-18.55 to 0.55
Sleep	5.12	0.23	-3.23 to 13.47	-3.33	0.54	-14.12 to 7.45
Auditory	-0.37	0.91	-6.66 to 5.91	-3.67	0.26	-10.12 to 2.79
Relaxation	-5.41	0.23	-14.33 to 3.50	-16.56	***0.001**	-26.78 to -6.34
Quality of life	0.14	0.97	-6.42 to 6.70	-6.42	0.09	-13.92 to 1.09
Emotional	0.72	0.86	-7.02 to 8.46	-10.11	**0.03**	-19.33 to -0.89

THQ = Tinnitus Handicap Questionnaire; TFI = Tinnitus Functional Index; Soc, emot & beh = social, emotional and behavioural effects subscale; SD = Standard Deviation; Grey shading = significant (uncorrected); * = significant after Bonferroni correction.

SUPPLEMENTARY FIGURES

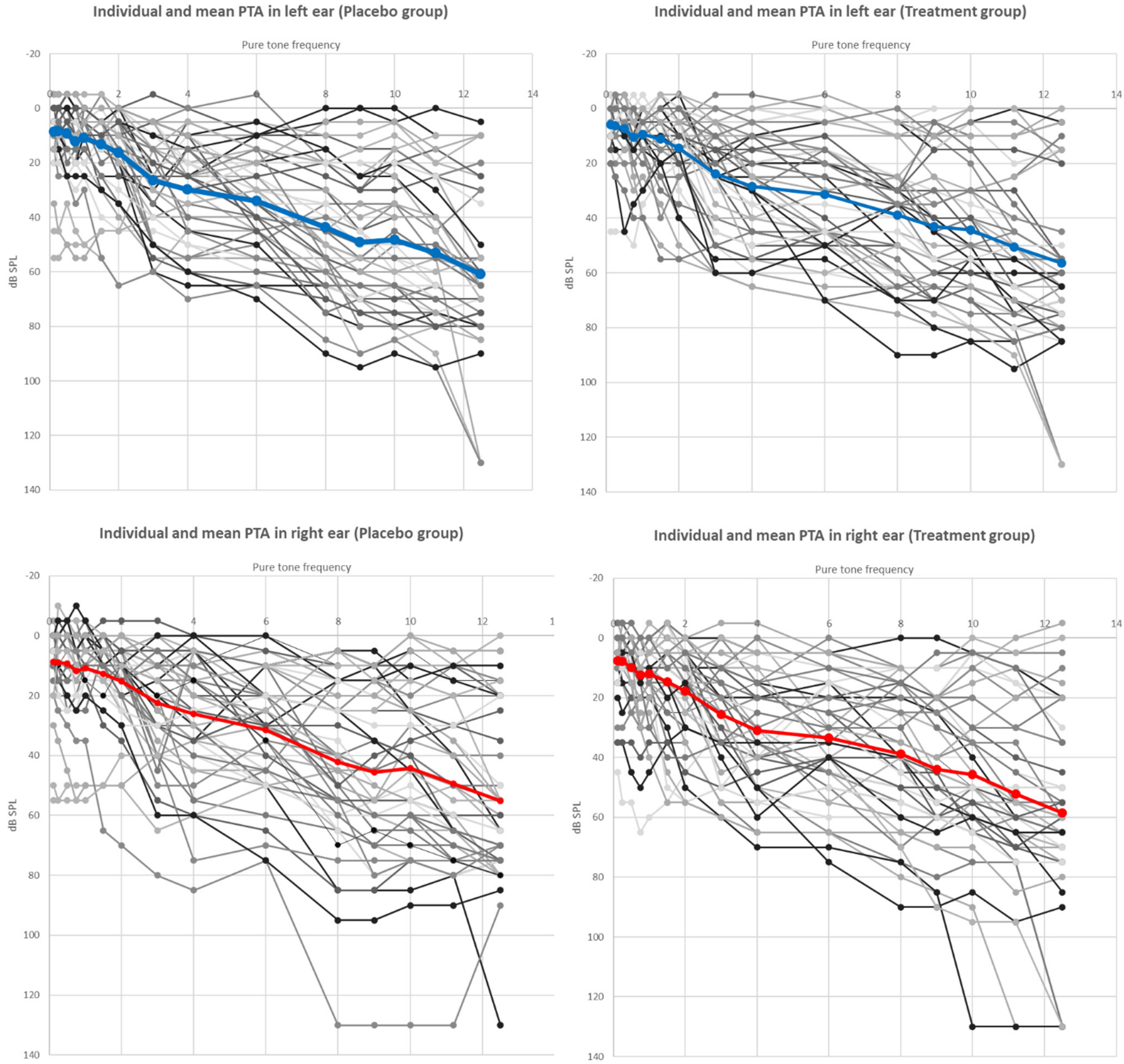

Supplementary Figure S1. Individual and average pure tone thresholds per ear per group.
